# Genome-wide identification and expression analysis of the SHAGGY-like kinase gene family in foxtail millet (*Setaria italica* L.)

**DOI:** 10.3389/fpls.2026.1852233

**Published:** 2026-06-01

**Authors:** Kangjie Qin, Yingying Sun, Yirong Wang, Zizhe Yang, Peixin Li, Zhenxia Su

**Affiliations:** 1 College of Life Sciences, Shanxi University, Taiyuan, China; 2 Biomedical and Health Laboratory in Shanxi Province, Shanxi University, Taiyuan, China

**Keywords:** adversity response, foxtail millet, gene function, GSK3, protein structure

## Abstract

**Introduction:**

Glycogen Synthase Kinase 3 (GSK3) is a family of highly conserved serine/threonine protein kinases originally identified in animals. Subsequently, homologous proteins with analogous functions were discovered in plants and designated as GSK3-like kinases or SHAGGY-like kinases (SKs). These kinases play pivotal roles in plants, including participation in growth and development, abiotic stress responses, and hormone signaling. However, the bioinformatics characteristics and protein functions of this gene family in foxtail millet remain poorly characterized.

**Methods:**

This study identified 9 SHAGGY-like kinase gene family members in the foxtail millet, which were classified into four subfamilies based on phylogenetic analysis. The functions of the gene family members were dissected by integrating bioinformatics analysis with quantitative real-time PCR (qRT-PCR).

**Results:**

Chromosomal localization analysis revealed uneven distribution of family members across chromosomes, prompting renaming based on their chromosomal order. Analysis of conserved domains, motifs, and gene structures demonstrated high conservation among subfamily members. Transcriptome analysis indicated active expression offoxtail millet SHAGGY-like kinase family members in stems and leaves. Stress treatment experiments showed that most foxtail millet SHAGGY-like kinase genes are responsive to abiotic stresses (drought, cold, NaCl) and exogenous hormones (ABA and MeJA).

**Conclusion:**

In summary, the phylogenetic and functional analysis of the foxtail millet SHAGGY-like kinase gene family provides insights into its role in stress responses and lays the foundation for future research aimed at enhancing the stress tolerance of foxtail millet.

## 1 Introduction

GSK3 is a class of evolutionarily conserved serine/threonine protein kinases identified in 1980. It was named for its ability to phosphorylate and thereby inhibit glycogen synthase (GS) (Embi et al., 1980). In mammals, two highly homologous isoforms—GSK3α (51 kDa) and GSK3β (47 kDa)—are encoded by distinct genes and share approximately 98% sequence identity in structure and function (Woodgett, 1990). Subsequent investigations have established GSK3 as a critical signaling node integrated into multiple cellular pathways, including PI3K/Akt, Wnt, Hedgehog, cyclic adenosine monophosphate (cAMP), DVL/GSK3/ISL1, MAPK, and transforming growth factor-beta (TGF-β) signaling (McCubrey et al., 2016; Dai et al., 2024). Furthermore, emerging evidence indicates that GSK3 can directly or indirectly phosphorylate voltage-gated sodium channels (e.g., Nav1.2 and Nav1.6) (James et al., 2015; Scala et al., 2018) and potassium channels (e.g., Kv4.2) (Aceto et al., 2019, 2020), thereby modulating the transmembrane transport of Na^+^ and K^+^. Consequently, GSK3 broadly orchestrates diverse biological processes in animals, encompassing metabolism, cell proliferation and differentiation, apoptosis, neuronal plasticity, and immune-inflammatory responses (Grimes and Jope, 2001; Doble and Woodgett, 2003; McCubrey et al., 2014).

In plants, although no direct homologs of the typical glycogen synthase kinase 3 exist, a class of functionally similar kinases is present. These share sequence homology with animal GSK3, particularly in the conserved kinase domain, but exhibit significant differences in their N- and C-terminal regulatory regions. To distinguish them, these plant kinases are often termed GSK3-like kinases. In *Arabidopsis thaliana*, they are collectively designated as SHAGGY-like kinases (SKs). Plant SHAGGY-like kinases play crucial roles in regulating growth and development, hormone signaling, and responses to abiotic stress (Tong et al., 2012; Cai et al., 2014; He et al., 2020; Xiang et al., 2025).

Numerous studies have established that SKs play pivotal roles in abiotic stress responses. In *Arabidopsis thaliana*, *AtSK21* has been identified as a key modulator of multiple abiotic stress responses. Primarily, *AtSK21* interacts with key heat stress transcription factors (Hsfs), specifically HsfA1s, to modulate plant thermotolerance (Luo et al., 2022). Furthermore, *AtSK21* functions as a molecular switch between salt stress signaling and growth by phosphorylating and modulating the activity of calcium sensors, *AtSK21* modulates the Salt Overly Sensitive (SOS) pathway by phosphorylating SOS2 and interacting with SOS3 and SCaBP8 (SOS3-like Calcium Binding Protein 8), thereby governing the transmembrane transport of sodium ions (Na^+^) (Li et al., 2020). Additionally, *AtSK21* is implicated in drought stress resistance; it directly interacts with and phosphorylates the stress-responsive NAC transcription factor RD26, a modification crucial for the transcriptional activation of drought-responsive genes (Jiang et al., 2019). Beyond *AtSK21*, the salt stress-activated SKs, *AtSK31*, influences nucleosome occupancy and chromatin accessibility by phosphorylating DEK3, thereby altering gene expression profiles to enhance salt tolerance (Waidmann et al., 2022). In rice, *OsSK21* interacts with the positive regulator of salt stress response, OsbZIP72, thereby negatively modulating salt tolerance (Liu et al., 2025); conversely, *OsSK21* knockout mutants exhibit enhanced salt stress tolerance (Koh et al., 2007). Additionally, the rice NAC (NAM, ATAF, CUC) transcription factor OsNAC016 interacts with and is phosphorylated by GSK2 (a negative regulator in the brassinosteroid (BR) pathway), consequently leading to the negative regulation of drought tolerance (Wu et al., 2022). Beyond these model species, members of the SKs have been shown to play significant regulatory roles in abiotic stress resistance in diverse crops and woody plants, including wheat (Aycan et al., 2022), potato (Huang et al., 2021), grapevine (Zeng et al., 2020), watermelon (Tian et al., 2026), tea plant (Yang et al., 2025), and rubber tree (Qiu et al., 2023).

In addition to the aforementioned important regulatory roles in abiotic stress, multiple studies have revealed that the SKs is extensively involved in the regulation of grain size, leaf development, floral organ development, embryogenesis, and root growth (Sun et al., 2015; Hu et al., 2018; Chen et al., 2020; Dong et al., 2020; Lyu et al., 2020; Hou et al., 2022; Wang et al., 2022; Qiu et al., 2025; Zhou et al., 2025), underscoring the functional diversity of this gene family during plant growth and developmental stages.

Foxtail millet, scientifically known as *Setaria italica* L., is an ancient crop that originated in China (Lu et al., 2009). It belongs to the genus *Setaria* within the Poaceae family and is an annual herbaceous plant. In China’s millennia-long agricultural history, it played an indispensable role, has been honored as “the foremost of the five grains.” The hulled seeds of foxtail millet are commonly referred to as “foxtail millet grains,” which possess high nutritional value. Throughout thousands of years of history, foxtail millet became the cornerstone of dryland farming in northern China due to its exceptional drought and nutrient-poor soil tolerance. This study conducted a comprehensive genome-wide characterization of the SKs in foxtail millet. According to the conventional nomenclature for the SHAGGY-like kinase genes in plant field, the members of the SHAGGY-like kinase genes in foxtail millet are collectively referred to as *SiSKs* hereafter. Based on the genome sequence, we identified nine SiSK proteins in foxtail millet. Their gene structures, chromosomal distributions, and conserved protein motifs were further analyzed. Additionally, expression profiles under various abiotic stresses were assessed to predict the functional roles of SiSKs. These findings provide a foundation for further investigation into the functions of *SiSKs* in plant responses to abiotic stress.

## 2 Materials and methods

### 2.1 Identification of the *SiSKs*

To identify SiSK genes, the protein sequences and.gff3 annotation files of foxtail millet were retrieved from Phytozome v13 (https://phytozome-next.jgi.doe.gov/). Gene identifiers of SHAGGY-like kinase gene family members for *Arabidopsis thaliana*, *Oryza sativa*, *Zea mays*, and *Triticum aestivum* were obtained from published literature. Subsequently, the corresponding SHAGGY-like kinase protein sequences for rice and *Arabidopsis* were retrieved from the Rice Genome Annotation Project database (http://rice.plantbiology.msu.edu/) and The *Arabidopsis* Information Resource (TAIR) database (https://www.arabidopsis.org/), respectively, while the maize and wheat SHAGGY-like kinase protein sequences were acquired from Phytozome v13. Detailed information on the SHAGGY-like kinase gene family members in the above four species is provided in **Supplementary Table 2**. Initially, multiple sequence alignment was performed against the foxtail millet protein sequence database using the BLAST function in TBtools (Chen et al., 2023). The candidate protein sequences obtained were further validated via NCBI Protein BLAST (https://blast.ncbi.nlm.nih.gov/Blast.cgi). Additionally, the definition of the Pkinase domain (Pfam: PF00069) was downloaded from InterPro (https://www.ebi.ac.uk/interpro/), and a Hidden Markov Model (HMM) was employed to verify the identified SiSK proteins. After deduplication, the sequences were submitted to NCBI-CDD (Marchler-Bauer et al., 2017; Lu et al., 2020; Wang et al., 2023) (https://www.ncbi.nlm.nih.gov/Structure/cdd/wrpsb.cgi) and the SMART (Letunic and Bork, 2026) (https://smart.embl.de/) online tool for domain screening. Phylogenetic evolutionary analyses incorporating *Arabidopsis* and rice were subsequently conducted. Ultimately, the confirmed protein and gene sequences of the SiSKs were obtained, and the genes were systematically renamed according to their chromosomal positions.

### 2.2 Phylogenetic analysis and physicochemical properties of the *SiSKs*

Full-length amino acid sequences of the SHAGGY-like kinase proteins from *Arabidopsis thaliana*, rice (*Oryza sativa*), maize (*Zea mays*), and foxtail millet (*Setaria italica*) were aligned using the ClustalW program implemented in MEGA (version 11.0) (Tamura et al., 2021). A phylogenetic tree was then constructed using the neighbor-joining (NJ) method with 1,000 bootstrap replicates.

The protein sequences of the SiSKs were uploaded to TBtools, and their physico-chemical properties were analyzed using the “Protein Parameter Calc” function.

### 2.3 Chromosomal localization analysis and subcellular localization analysis of the *SiSKs*

The chromosomal positions of *SiSKs* were obtained from the NCBI database (https://www.ncbi.nlm.nih.gov/), while the lengths of individual foxtail millet chromosomes were retrieved from the UCSC database (https://genome.ucsc.edu/). Chromosomal localization visualization was subsequently performed using TBtools.

The protein sequences of the SiSKs were submitted to WoLF PSORT (https://wolfpsort.hgc.jp/) for subcellular localization prediction.

### 2.4 Genetic structure and analysis of conserved motifs in the *SiSKs*

The gene structure of the *SiSKs* was analyzed and visualized using the Gene Structure Display Server (GSDS 2.0, https://gsds.gao-lab.org/Gsds_help.php) (Hu et al., 2015). Conserved motifs were identified with the MEME (Bailey et al., 2006) online tool (https://meme-suite.org/meme/tools/meme) using a parameter of 10 motifs, and the results were visualized via TBtools.

### 2.5 Analysis of Cis-acting regulatory elements in the promoters of the SiSK genes

To investigate the cis-acting regulatory elements in the putative SiSK genes, a BLAST search was performed against the foxtail millet genome using the corresponding gene IDs to retrieve their promoter sequences (2,000 bp upstream of the translation start codon “ATG”). The extracted promoter sequences were subsequently analyzed using the PlantCARE (Lescot et al., 2002) online tool (https://bioinformatics.psb.ugent.be/webtools/plantcare/html/) to identify potential cis-acting regulatory elements.

### 2.6 Analysis of abiotic stress treatment and expression patterns

Plump seeds of the foxtail millet cultivar Yugu1 were selected and germinated, then transferred to hydroponic boxes for two weeks of cultivation. Seedlings were subsequently subjected to treatments simulating various abiotic stresses: 20% PEG6000 (Pan et al., 2016) (drought stress), 150 mM NaCl (salt stress), 100 µM ABA (Zhao et al., 2019) (hormonal stress), 100 µM MeJA (hormonal stress), and low temperature at 4 °C (Fan et al., 2021) (cold stress). Leaf samples were collected and flash-frozen at five time points (0 h, 4 h, 8 h, 12 h, and 24 h) post-treatment. Total RNA was extracted from samples derived from three distinct foxtail millet (*Setaria italica*) plants, and subsequently reverse-transcribed into cDNA. Quantitative real-time PCR (qRT-PCR) was performed using the SYBR Green fluorescence method with three technical replicates for each sample to minimize experimental error. The *Setaria italica Actin7* gene was used as an internal reference gene for normalization.

Data are presented as the mean ± standard error of the mean (SEM) and were analyzed and visualized using GraphPad Prism software. Normality and log-normality were assessed prior to analysis. Data conforming to a normal distribution with homogeneity of variance were subjected to ordinary one-way analysis of variance (ANOVA). When data met the assumption of normality but violated homogeneity of variance, Brown-Forsythe or Welch’s ANOVA tests were employed. For data that did not follow a normal distribution, the Kruskal-Wallis test was used. Statistically significant differences among groups were determined, and results with different letters indicate significant differences (*P* < 0.05). Detailed information on the primers used in this study is provided in **Supplementary Table 1**.

### 2.7 Secondary and tertiary structure analysis of the SiSK proteins

The secondary structures of proteins in the SiSKs were predicted using the SOPMA (https://npsa.lyon.inserm.fr/cgi-bin/npsa_automat.pl?page=/NPSA/npsa_sopma.html) server. Tertiary structures were retrieved from the SWISS-MODEL (https://swissmodel.expasy.org/) repository using default parameter settings. Additionally, the tertiary structures of these proteins were inspected via the PyMOL Molecular Graphics System, Version 3.0 Schrödinger, LLC (https://pymol.org/support.html?#citing).

### 2.8 Transcriptome analysis of the SiSK genes

Transcriptomic data for the identified SiSKs genome were downloaded (http://111.203.21.71:8000/multi-omics/transcriptome.html) (Li et al., 2025), and the transcriptional expression levels of these family members were subsequently analyzed. A grouped clustering heatmap was generated using TBtools software to visualize the expression patterns.

### 2.9 Collinearity analysis

Synteny analysis of the SiSKs genome was performed using the methodology described by Dr. Chen’s team and implemented with TBtools (Chen et al., 2022). Furthermore, comparative synteny analysis was performed among the SHAGGY-like kinase gene families of foxtail millet, rice, and maize. The results of these syntenic relationships were visualized using TBtools.

## 3 Results

### 3.1 Genetic family identification and physicochemical properties analysis of the *SiSKs*

Following redundancy removal, a total of nine SiSK genes were identified and were renamed *SiSK1*–*SiSK9* based on phylogenetic analysis (**Table 1**). The results revealed that, with the exception of *SiSK7*, which comprises only 119 amino acid residues with an isoelectric point (pI) below 7, indicating an acidic nature, all remaining SiSKs contain more than 400 amino acid residues and exhibit pI values above 7, suggesting a basic nature.

**Table 1:** Basic information of genes.

Gene name	Gene ID	Number of amino acid	Molecular weight	Theoretical pI	Instability index	Aliphatic index	Grand average of hydropathicity
SiSK1	Seita.1G023300	414	46033.29	8.71	43.06	86.30	-0.145
SiSK2	Seita.3G052100	410	46553.81	8.55	40.89	88.93	-0.263
SiSK3	Seita.4G177000	402	45028.22	8.65	48.24	87.59	-0.166
SiSK4	Seita.5G029400	408	46406.79	8.65	40.68	90.00	-0.221
SiSK5	Seita.5G060800	408	46051.48	8.90	33.80	89.56	-0.262
SiSK6	Seita.5G145300	406	45402.49	8.43	46.46	83.65	-0.235
SiSK7	Seita.7G053200	119	13291.47	6.48	25.93	107.31	0.168
SiSK8	Seita.9G017600	426	48494.67	8.56	47.54	82.37	-0.365
SiSK9	Seita.9G349600	457	51621.42	8.56	38.99	82.08	-0.406

### 3.2 Phylogenetic analysis of the *SiSKs*

**Figure 1:**
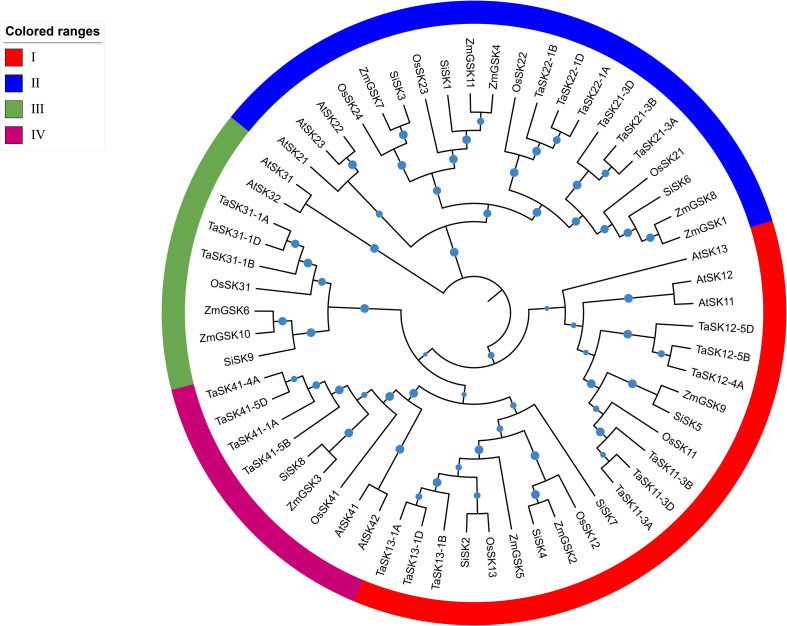
Phylogenetic analysis of GSK3 proteins in *Arabidopsis*, rice, maize, wheat, and foxtail millet.

To accurately resolve the evolutionary relationships among SiSK proteins, we performed a neighbor-joining (NJ) phylogenetic analysis of SHAGGY-like kinase proteins from *Arabidopsis thaliana*, *Oryza sativa*, *Zea mays*, *Triticum aestivum*, and *Setaria italica* (**Figure 1**). All proteins were partitioned into four clades (I–IV). Moreover, the greater number of SiSKs and their higher sequence similarity to OsSKs than to AtSKs indicate that SiSKs are more closely related to OsSKs than to AtSKs. Consistent with the phylogenetic tree topology, SiSKs clustered more closely with their homologs from OsSKs, ZmSKs, and TaSKs (monocots) than with AtSKs (dicots). These results suggest that SHAGGY-like kinase genes are not strictly conserved across species and have diversified during evolution.

### 3.3 Chromosomal localization analysis of the *SiSKs*

**Figure 2:**
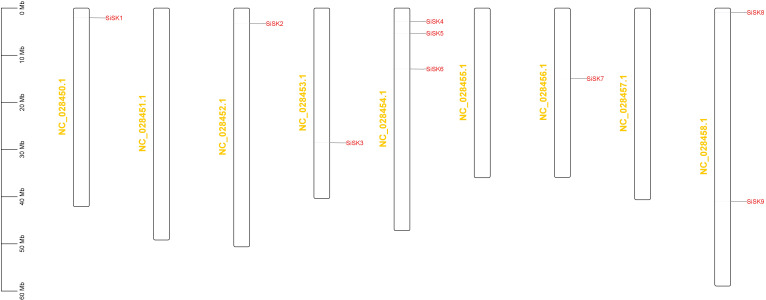
*SiSKs* chromosome localization analysis.

The distribution of *SiSKs* across the nine chromosomes of the foxtail millet genome is non-uniform (**Figure 2**). No genes were localized on chromosomes 2, 6, and 8. Three genes were mapped to chromosome 5, while two genes were identified on chromosome 9. The remaining four SiSKs were each singly and uniformly distributed on chromosomes 1, 3, 4, and 7.

### 3.4 Analysis of gene structure, conserved motifs, and conserved domains in the *SiSKs*

**Figure 3:**
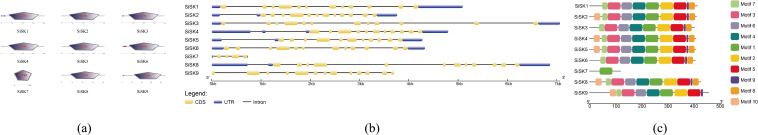
Analysis of the SiSK gene's conserved domain, gene structure, and conserved motifs. **(A)** Conserved domain of SiSKs. **(B)** Exon–intron structures of *SiSKs*. The full-length mRNA sequences of *SiSKs* were analyzed and displayed. Coding sequences (CDSs) are represented by yellow boxes, untranslated regions (UTRs) are shown as blue boxes, and introns are represented by blank lines. The scale at the bottom represents lengths. **(C)** Conserved motif analysis of SiSKs. Motifs were designated 1–10 and distinguished with different colors.

Analysis using the SMART online tool confirmed that *SiSKs* contains a typical serine/threonine kinase (STKc) catalytic domain (**Figure 3A**). To gain deeper insight into the structural features of the predicted SiSK genes, we determined their intron-exon and UTR organization (**Figure 3B**). Structural analysis revealed that, except for *SiSK7* (which possesses four introns), the coding regions of all other SiSK genes are interrupted by 10–12 introns. The SiSK7 sequence is significantly shorter in length. The UTR regions of *SiSK9* have not been determined, whereas the remaining SiSK genes possess UTR annotations in the genome. Overall, the four groups of the SHAGGY-like kinase gene family—particularly Groups I, II, and IV—exhibit highly similar gene structures in terms of both the number and length of exons. MEME was used to analyze conserved motifs among nine SiSK proteins (**Figure 3C**). A total of 10 putative conserved motifs, each 6–50 amino acid residues in length, were identified. All members contained motif 1, and motifs 2–9 were present in nearly all SiSK members except SiSK7. SiSK7 lacks several conserved motifs present in other SiSKs, Consequently, we tentatively classify SiSK7 as an atypical or truncated candidate gene. Due to its structural incompleteness, its precise relationship with the core SHAGGY-like kinase family remains to be elucidated and requires further experimental validation.

### 3.5 Analysis of Cis-acting regulatory elements in the promoters of the SiSK genes

**Figure 4:**
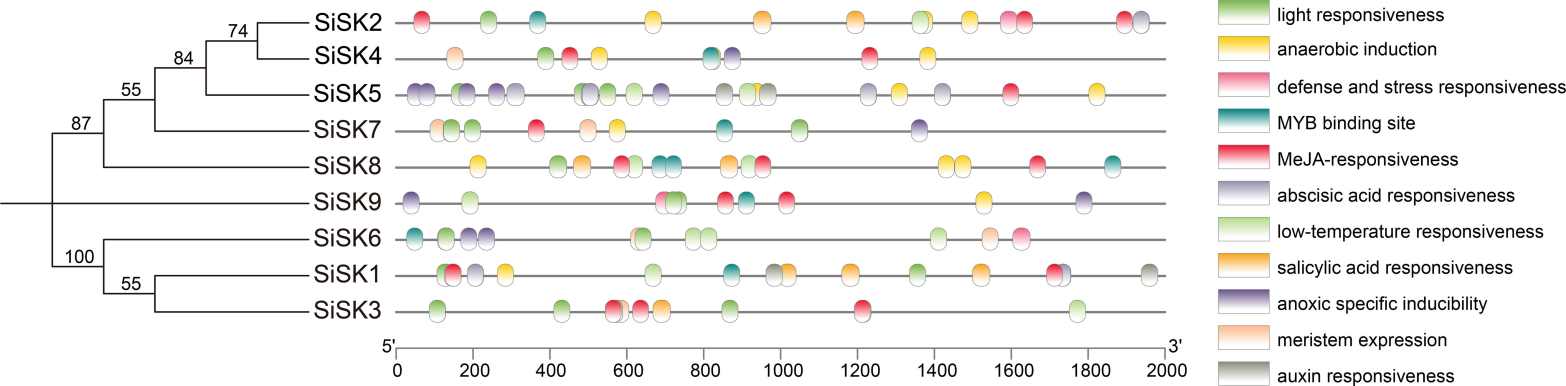
Identification of cis-regulatory elements in the *SiSKs* promoter.

To further elucidate the regulatory mechanisms of SiSK genes, we examined their upstream promoter sequences (2000 base pairs upstream of the coding region) and predicted cis-acting elements using PlantCARE (**Figure 4**). A total of 11 distinct cis-acting elements were identified within these promoter regions. Notably, cis-regulatory elements associated with methyl jasmonate (MeJA) response and light responsiveness were found in the promoters of all SiSK genes. Additionally, cis-acting elements implicated in abscisic acid (ABA) response were detected in eight SiSK gene promoters, specifically SiSK1, 2, 3, 5, 6, 7, 8, and 9. Intriguingly, all SiSK gene promoters contained multiple cis-regulatory elements related to MeJA response, suggesting a pronounced hormonal responsiveness to MeJA. All cis-elements identified in this study are involved in hormone signaling, stress responses, and represent crucial transcription factor binding sites.

### 3.6 Subcellular localization analysis of the *SiSKs*

Using WoLF PSORT, a score was computed for each potential subcellular localization on the basis of all features extracted from the SiSK protein sequences, including the signal peptide, transmembrane regions, and nuclear localization signal (NLS). The putative locations were then ranked from highest to lowest score, and the most probable localization prediction was reported together with its confidence. The final results indicate that, with the exception of SiSK3, all other SiSK proteins possess multiple potential subcellular localizations, and the majority are predicted with high likelihood to reside in the cytoplasm (**Table 2**).

**Table 2:** Subcellular localization prediction results.

Query protein	CELLO prediction				
SiSK1	cytosol:5	cytosolskeleton:5	Vascular membrane:1.5	chloroplast:1	nucleus:1
SiSK2	chloroplast:6	cytosol:5	mitochondrion:2	Plasma membrane:1	
SiSK3	cytosolskeleton:14				
SiSK4	cytosol:10	cytosolskeleton:2	nucleus:1	extracellular:1	
SiSK5	cytosol:9	nucleus:2	mitochondrion:2	peroxisome:1	
SiSK6	cytosol:8	nucleus:2	cytosolskeleton:2	Golgi apparatus:1	
SiSK7	chloroplast:9	cytosol:2	extracellular:2	Endoplasmic reticulum:1	
SiSK8	mitochondrion:7	chloroplast:3.5	nucleus:2	Plasma membrane:1	
SiSK9	mitochondrion:6.5	nucleus:4	chloroplast:2	cytosol:1	

### 3.7 Prediction of secondary and tertiary structures of the *SiSKs*

Protein secondary structure prediction of the SiSK family members revealed that all proteins contain three primary types of secondary structures: alpha helix (Hh), extended strand (Ee), and random coil (Cc) (**Table 3**). The proportional distribution of amino acids contributed by each secondary structure type was highly similar among the SiSK proteins. Notably, the extended strand (Ee) exhibited particularly consistent amino acid composition across all members, with the exception of SiSK7. Furthermore, the number of amino acids attributed to each secondary structure was also extremely comparable among the majority of the SiSK proteins. These findings demonstrate a high degree of structural conservation within the SiSK proteins.

**Table 3:** Prediction of secondary structures of the *SiSK* protein.

	Alpha helix(Hh)	Extended strand(Ee)	Random coil(Cc)
SiSK1	143 is 34.54%	55 is 13.29%	216 is 52.17%
SiSK2	152 is 37.07%	50 is 12.20%	208 is 50.73%
SiSK3	142 is 35.32%	50 is 12.44%	210 is 52.24%
SiSK4	166 is 40.69%	52 is 12.75%	190 is 46.57%
SiSK5	159 is 38.97%	51 is 12.50%	198 is 48.53%
SiSK6	141 is 34.73%	54 is 13.30%	211 is 51.97%
SiSK7	41 is 34.45%	18 is 15.13%	60 is 50.42%
SiSK8	146 is 34.27%	55 is 12.91%	225 is 52.82%
SiSK9	148 is 32.39%	53 is 11.60%	256 is 56.02%

**Figure 5:**
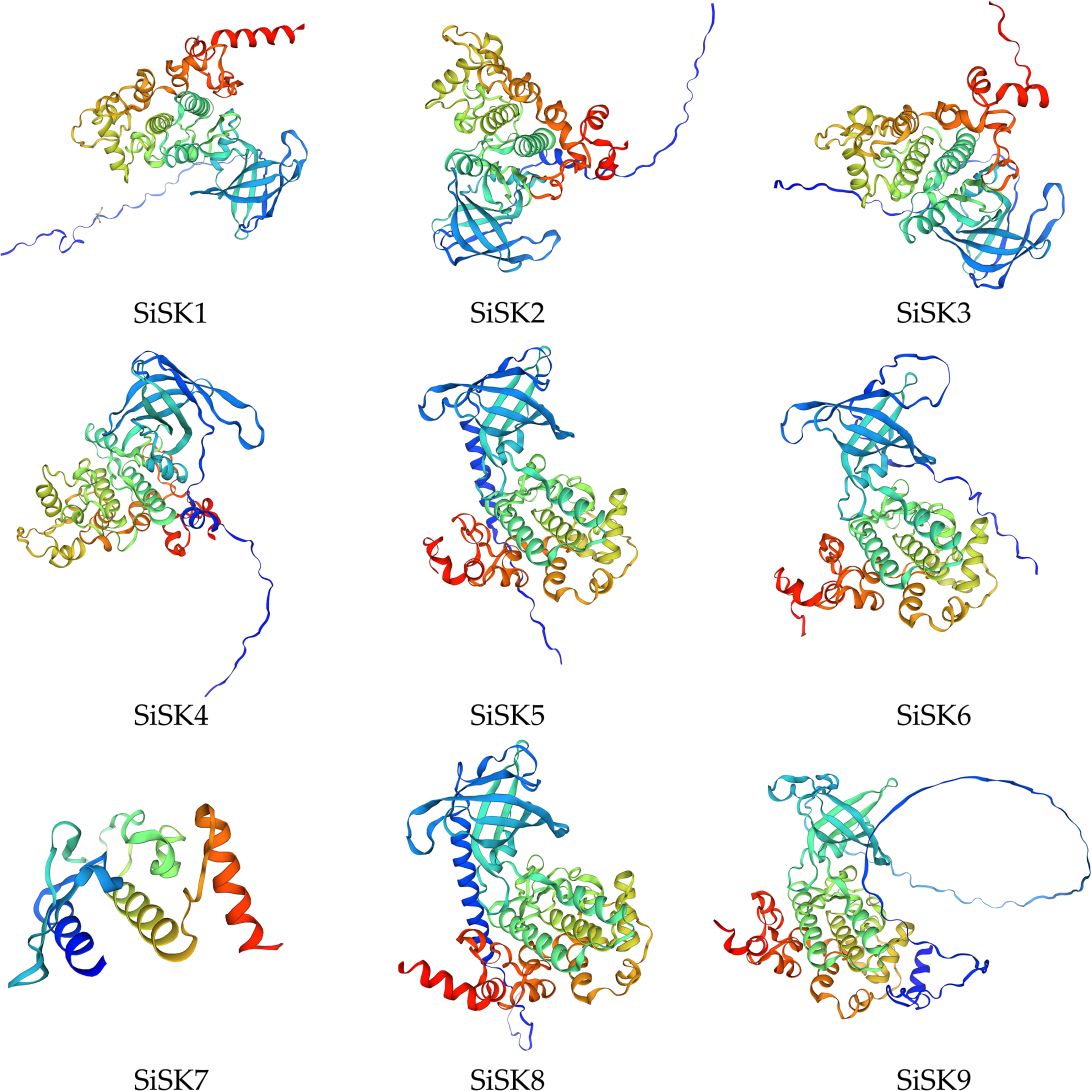
Prediction of the tertiary structures of the SiSK proteins.

Prediction of the tertiary structure of the SiSK proteins revealed that each protein contains a structurally conserved domain with a highly similar spatial conformation, which is likely to play a crucial functional role. Overall, the homology models of these proteins provide a preliminary structural basis for further elucidating the molecular functions of the SiSKs (**Figure 5**).

### 3.8 Collinearity analysis of foxtail millet, *Arabidopsis*, rice, and maize

**Figure 6:**
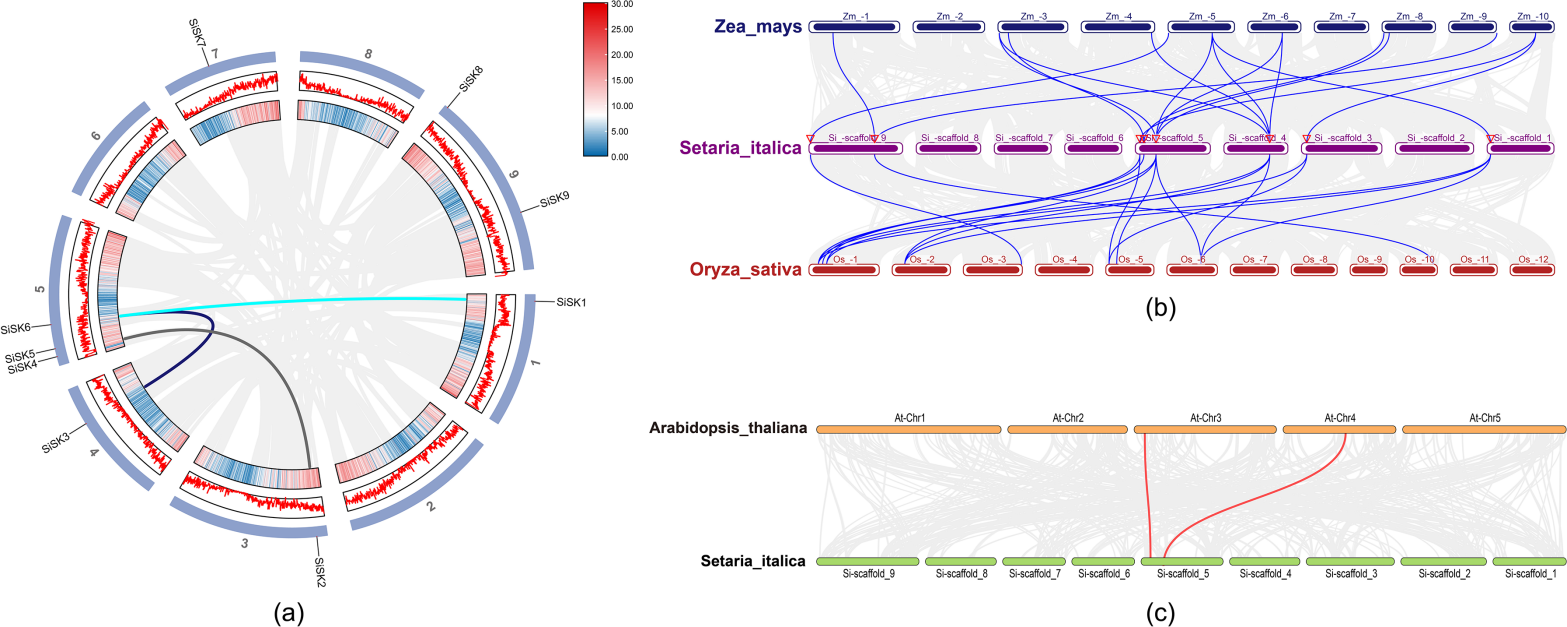
Collinearity analysis results. **(a)** Collinearity analysis of the foxtail millet genome. **(b)** Collinearity analysis among the genomes of foxtail millet, maize, and wheat. **(c)** Collinearity analysis between the foxtail millet and Arabidopsis thaliana genomes.

Synteny analysis across different species serves as a method to investigate their evolution and phylogenetic relationships. To further elucidate the phylogenetic mechanisms of SHAGGY-like kinase genes, we performed a comparative synteny analysis between foxtail millet and three representative species: one dicotyledonous plant (*Arabidopsis thaliana*) and two monocotyledonous plants (*Oryza sativa* and *Zea mays*) (**Figure 6**). The results revealed that the degree of synteny between foxtail millet and the monocot genomes was greater than that between foxtail millet and the dicot genome. Individual homologous genes exhibited one-to-many or many-to-one orthologous relationships. Notably, two SiSK genes (*SiSK5* and *SiSK6*) showed syntenic associations with all three selected species, suggesting that these genes within the SHAGGY-like kinase gene family may have played significant roles during evolution.

### 3.9 Expression profiles analysis of the *SiSKs*

**Figure 7:**
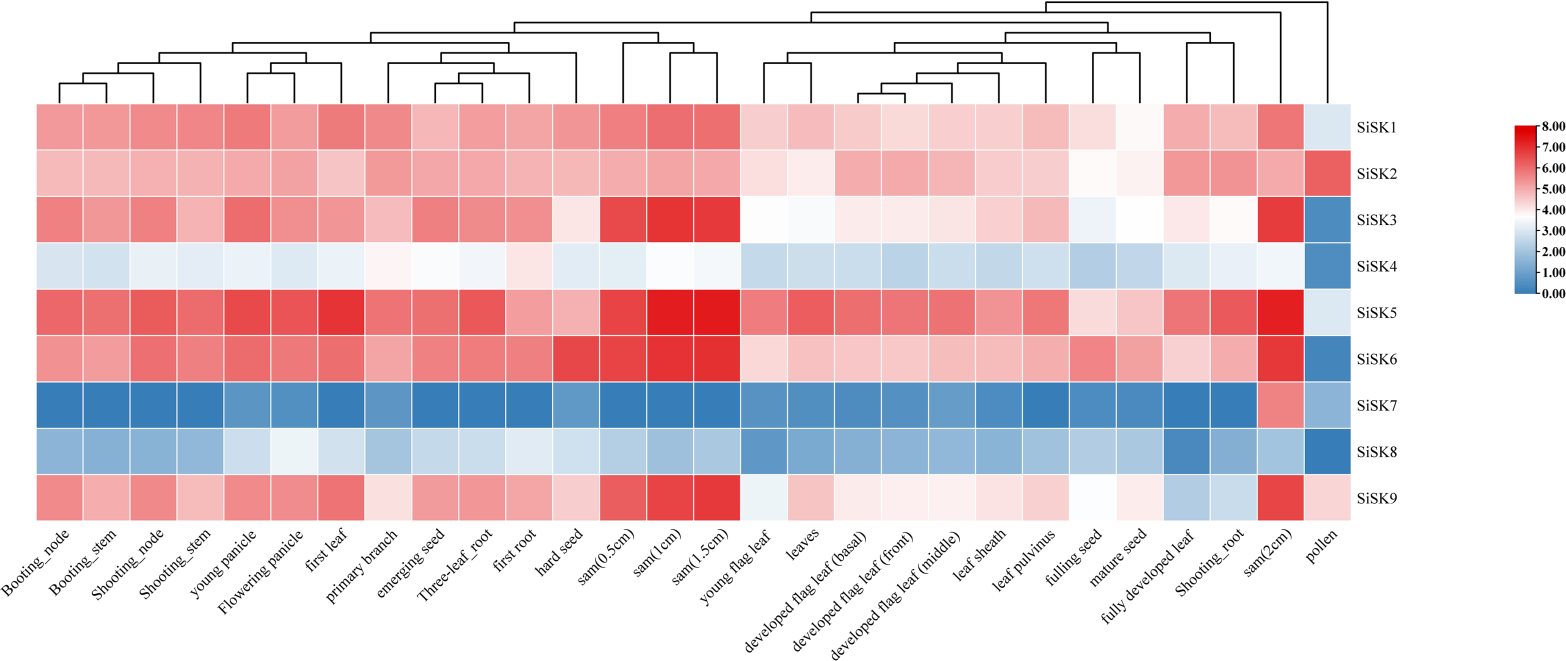
Transcriptome analysis results visualized as heatmaps.

Transcriptome profiling of the SiSKs revealed substantial variation in expression levels among members (**Figure 7**). Compared with other tissues, members of SHAGGY-like kinase Groups II (*SiSK1*, *SiSK3* and *SiSK6*) and III (*SiSK9*) exhibited higher expression than those of Groups I and IV, and the majority of family members showed relatively high and stable expression in young panicles during the booting stage. Members of Groups II and III were expressed across nearly all tissues throughout the developmental stages of foxtail millet. By contrast, *SiSK4*, *SiSK7*, and *SiSK8* displayed consistently low expression across all tissues, a pattern consistent with their likely roles as inducible genes that are activated only upon exposure to external stimuli.

### 3.10 Analysis of the expression patterns of the *SiSKs* under abiotic stress conditions

#### 3.10.1 Expression pattern analysis of the *SiSKs* under drought stress

**Figure 8:**
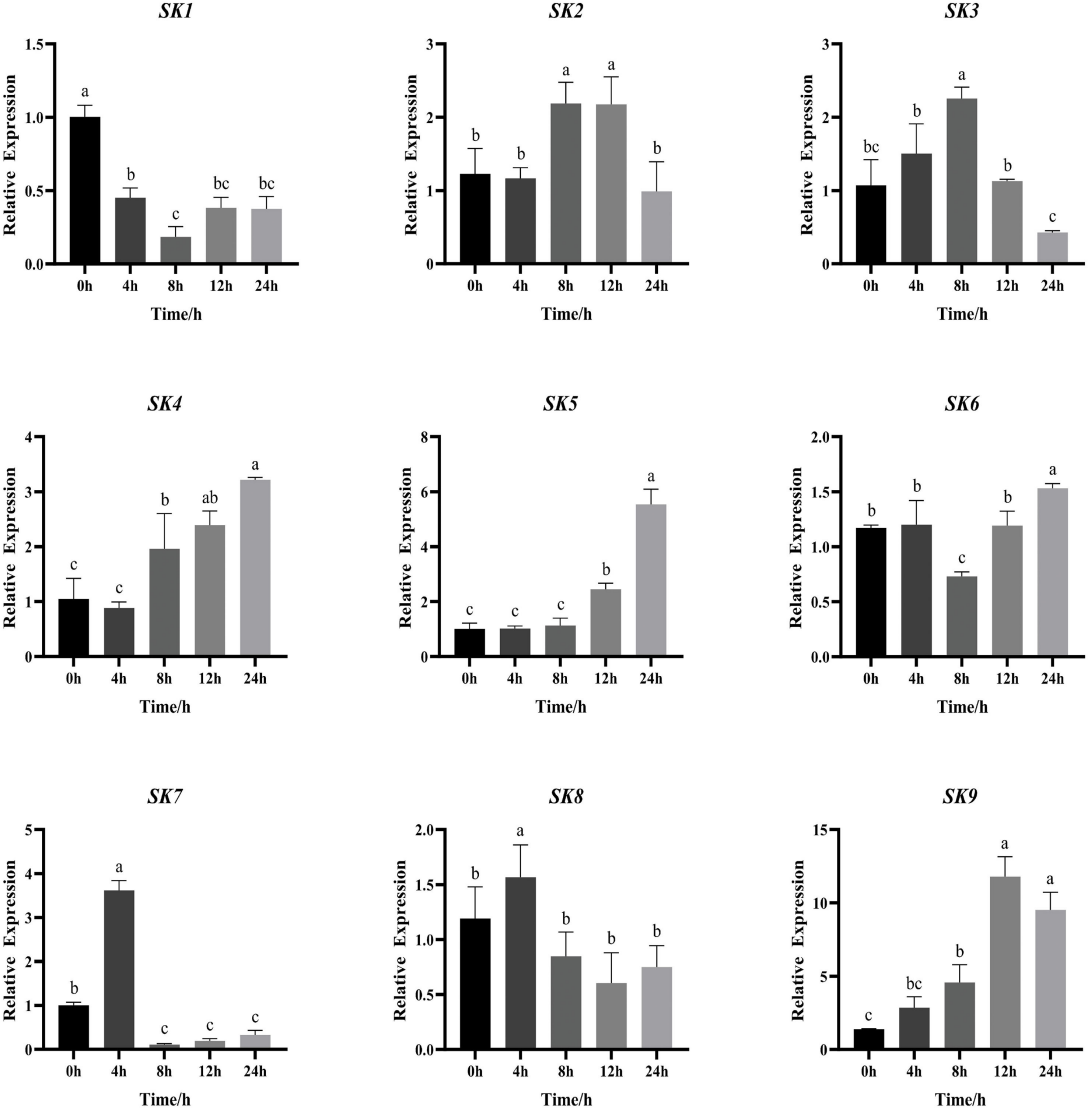
Expression patterns of the *SiSKs* under drought stress. Different lowercase letters indicate statistically significant differences (P<0.05).

Within 24 hours following simulated drought stress treatment, distinct expression profiles of the *SiSKs* were observed in foxtail millet. Specifically, *SiSK1* exhibited a significant and sustained downregulation. In contrast, *SiSK2*, *SiSK3*, *SiSK7* and *SiSK8* showed an initial significant upregulation followed by a marked downregulation. Meanwhile, *SiSK4*, *SiSK5* and *SiSK9* displayed a consistent and significant upregulation, suggesting their potential role as positive regulators. Notably, *SiSK6* exhibited a transient downregulation followed by a significant upregulation (**Figure 8**). Collectively, these results indicate that *SiSK1*, *SiSK4*, *SiSK5* and *SiSK9* are likely involved in the drought stress response in foxtail millet, whereas the functional relevance of *SiSK2*, *SiSK3*, *SiSK6*, *SiSK7* and *SiSK8* warrants further investigation.

#### 3.10.2 Analysis of the expression patterns of the *SiSKs* under low temperature stress

**Figure 9:**
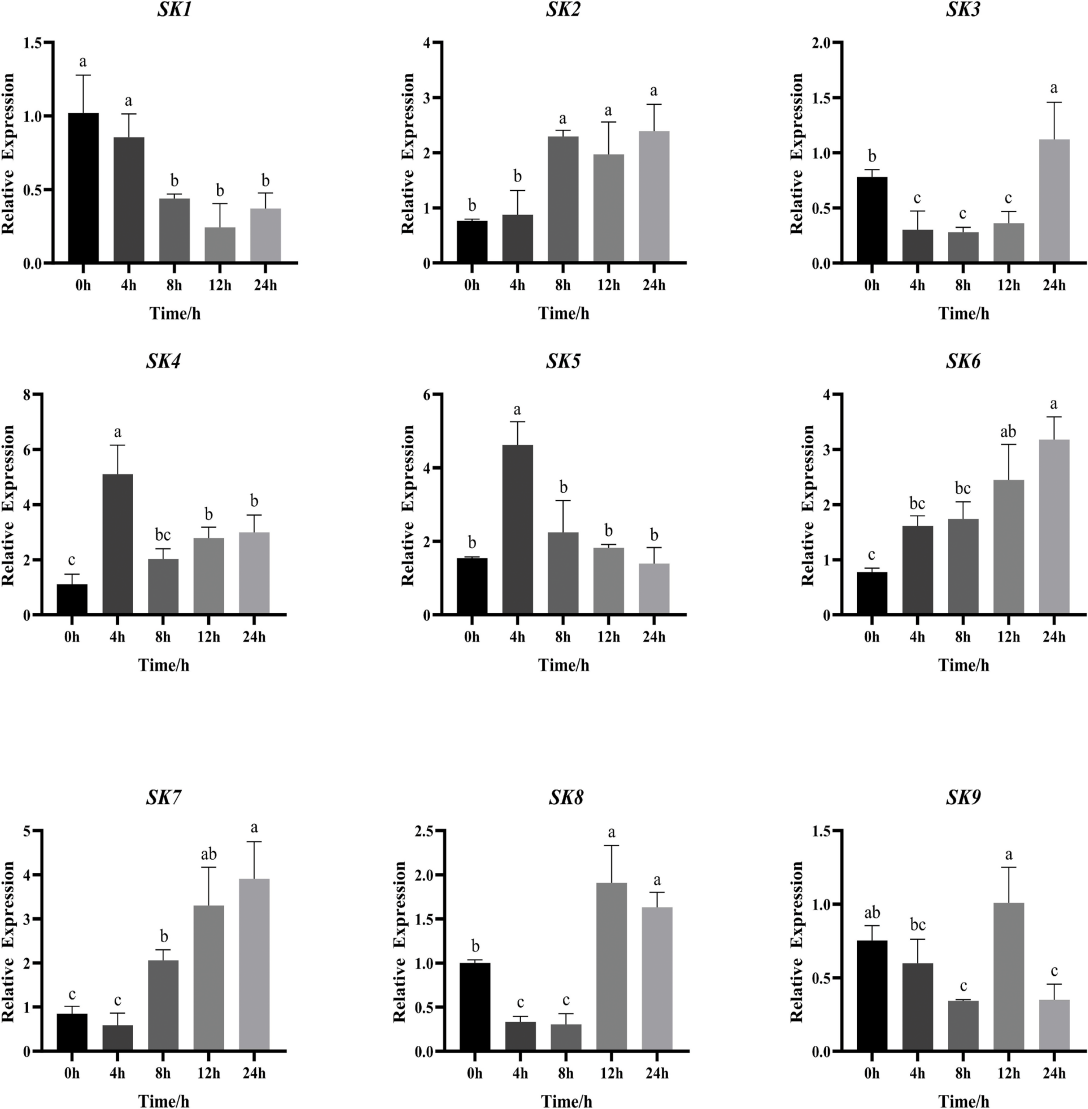
Expression patterns of the *SiSKs* under low-temperature stress. Different lowercase letters indicate statistically significant differences (P<0.05).

Following exposure to simulated low-temperature stress for 24 hours, the *SiSKs* in foxtail millet exhibited distinct expression dynamics. The transcript level of *SiSK1* demonstrated a consistent and significant decline throughout the treatment. In contrast, *SiSK2*, *SiSK6* and *SiSK7* showed stable and significant upregulation during the entire stress period. Furthermore, cis-acting element analysis revealed the presence of multiple low-temperature-responsive elements in the promoter region of *SiSK6*, corroborating the quantitative expression data. *SiSK3* and *SiSK8* exhibited a biphasic pattern characterized by an initial significant decrease followed by a significant increase. Conversely, *SiSK4* and *SiSK5* displayed an opposite trend, with an initial significant upregulation followed by a marked downregulation. Notably, *SiSK9* showed a triphasic expression profile: its transcript abundance significantly decreased, subsequently increased, and finally declined again within the 24-hour timeframe (**Figure 9**). Collectively, these findings suggest that *SiSK1*, *SiSK2*, *SiSK6* and *SiSK7* are potentially involved in the low-temperature stress response in foxtail millet, whereas the functional relevance of *SiSK3*, *SiSK4*, *SiSK5*, *SiSK8* and *SiSK9* warrants further investigation.

#### 3.10.3 Expression pattern analysis of the *SiSKs* under salt stress

**Figure 10:**
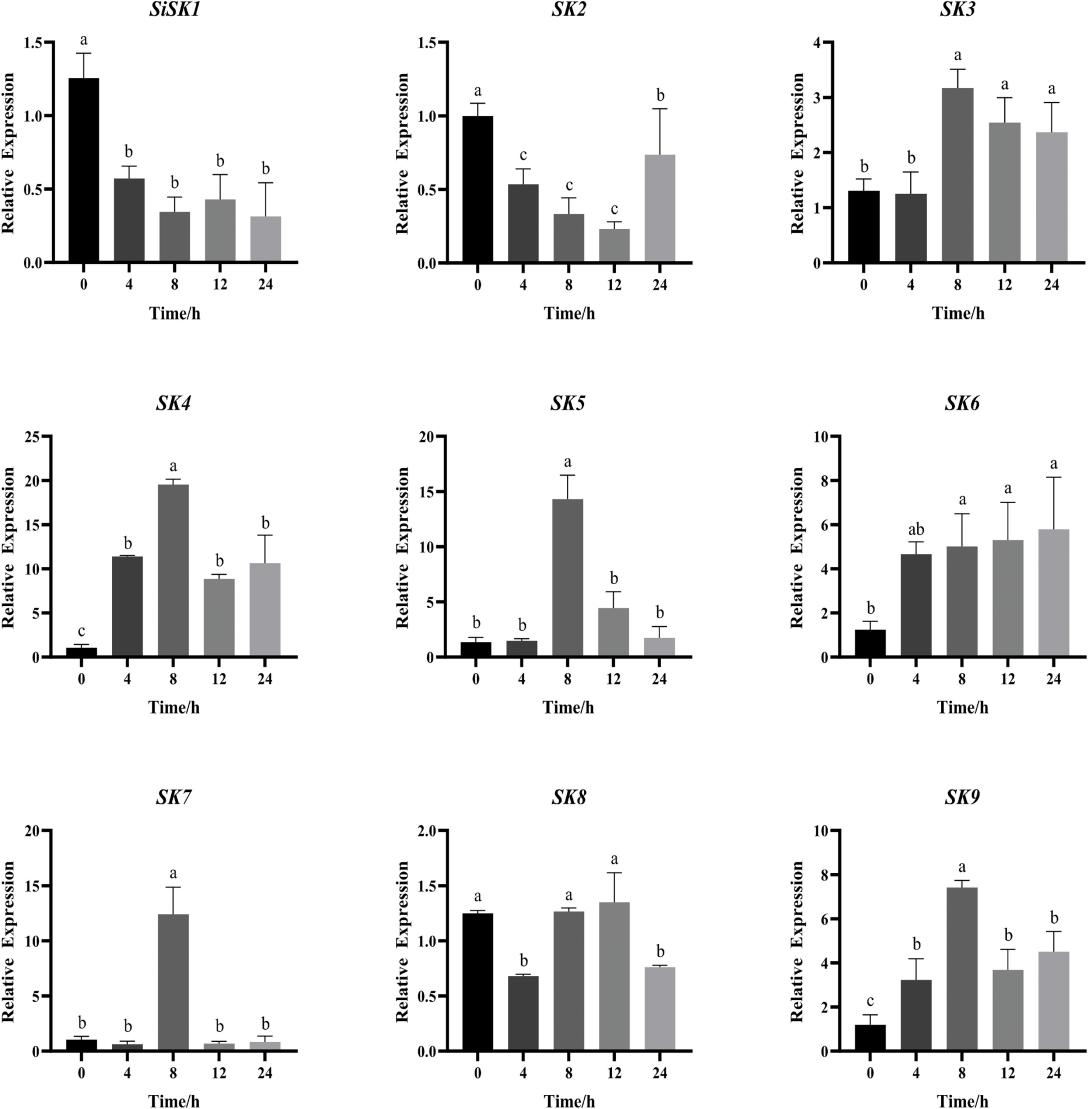
Expression patterns of the *SiSKs* under salt stress. Different lowercase letters indicate statistically significant differences (P<0.05).

Within 24 hours following simulated salt stress treatment, distinct expression profiles of the *SiSKs* were observed in foxtail millet. Specifically, *SiSK1* exhibited a significant and sustained downregulation. In contrast, *SiSK2* and *SiSK8* showed an initial significant downregulation followed by a marked upregulation; however, *SiSK8* subsequently experienced a significant secondary downregulation. Meanwhile, *SiSK3* and *SiSK6* displayed a consistent and significant upregulation throughout the treatment. Conversely, *SiSK4*, *SiSK5*, *SiSK7* and *SiSK9* exhibited an initial significant upregulation followed by a marked downregulation (**Figure 10**). Collectively, these results indicate that *SiSK1*, *SiSK3* and *SiSK6* are likely involved in the salt stress response in foxtail millet, whereas the functional relevance of *SiSK2*, *SiSK4*, *SiSK5*, *SiSK7*, *SiSK8* and *SiSK9* warrants further investigation.

#### 3.10.4 Analysis of the expression patterns of the *SiSKs* under abscisic acid regulation

**Figure 11:**
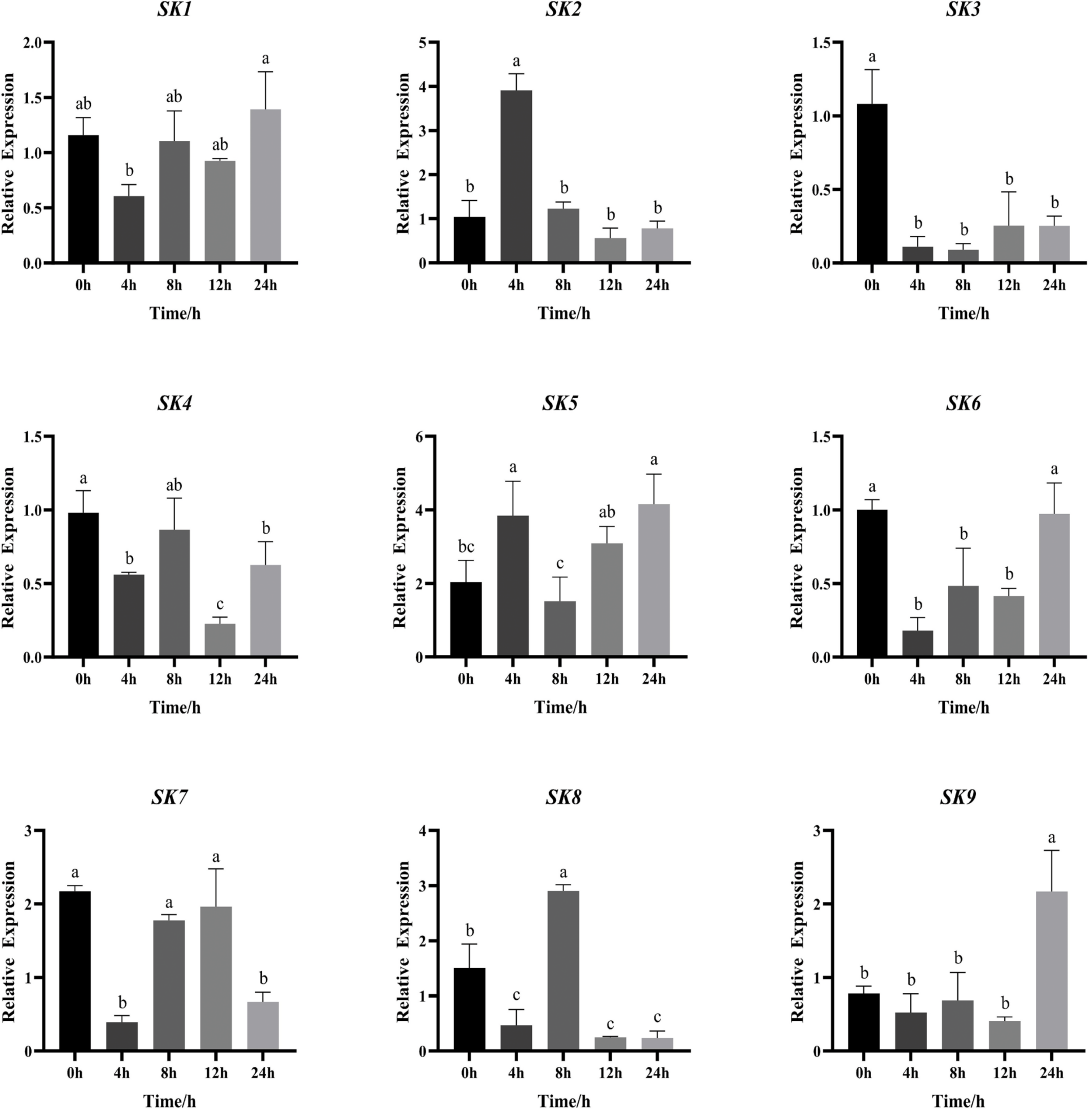
Expression patterns of the *SiSKs* under ABA regulation. Different lowercase letters indicate statistically significant differences (P<0.05).

Dynamic expression profiles of *SiSKs* were observed in hydroponically grown foxtail millet within 24 hours following abscisic acid (ABA) treatment. Specifically, *SiSK1*, *SiSK4* and *SiSK6* exhibited an initial significant downregulation followed by a marked upregulation. In contrast, *SiSK2* showed an initial significant upregulation followed by a notable decline. The transcript level of *SiSK3* demonstrated a consistent and significant decrease throughout the observation period. Intriguingly, *SiSK5* displayed a triphasic pattern characterized by an initial upregulation, a subsequent downregulation, and a final significant increase. Similarly, *SiSK7* and *SiSK8* exhibited complex dynamics, with transcript levels initially decreasing, then rising, and ultimately declining again. Notably, the transcript abundance of *SiSK9* showed a sustained and significant increase over the entire 24-hour period (**Figure 11**). Furthermore, cis-acting element analysis revealed the presence of multiple ABA-responsive elements in the promoters of *SiSK1* and *SiSK5*, corroborating the quantitative expression data. Collectively, these results indicate that *SiSK3* and *SiSK9* are likely involved in the ABA response in foxtail millet, whereas the functional relevance of the remaining *SiSKs* warrants further investigation.

#### 3.10.5 Expression pattern analysis of the *SiSKs* under methyl jasmonate (MeJA) regulation

**Figure 12:**
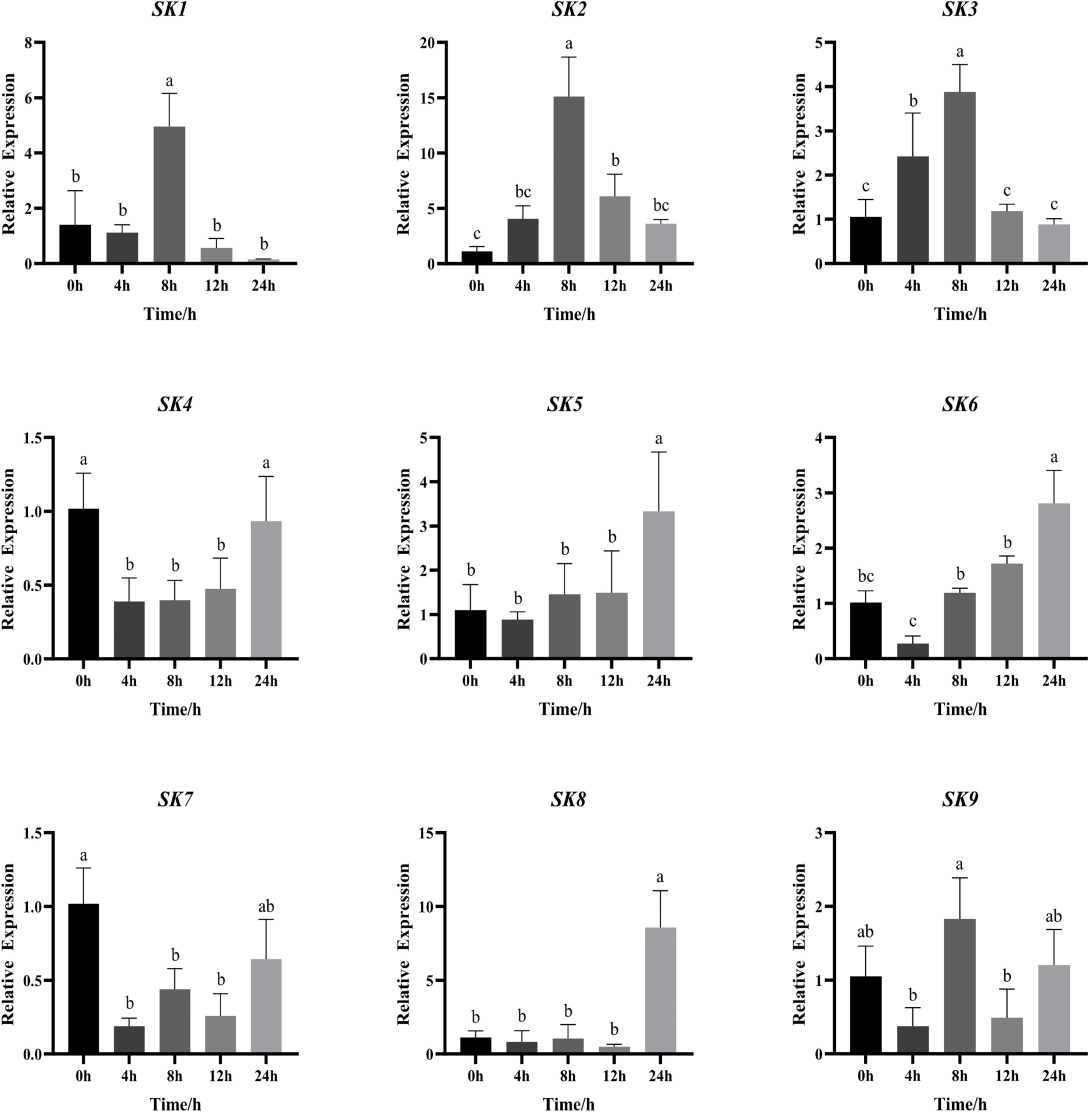
Expression patterns of the *SiSKs* under MeJA regulation. Different lowercase letters indicate statistically significant differences (P<0.05).

Within 24 hours following methyl jasmonate (MeJA) treatment in hydroponically grown foxtail millet, distinct expression profiles of the *SiSKs* were observed (**Figure 12**). Specifically, *SiSK1*, *SiSK2*, *SiSK3* and *SiSK9* exhibited an initial significant upregulation followed by a marked downregulation. In contrast, *SiSK4* showed an initial significant downregulation followed by a notable upregulation. Meanwhile, *SiSK5*, *SiSK6* and *SiSK8* displayed a sustained and significant upregulation throughout the observation period, whereas *SiSK7* showed a persistent downregulation. Furthermore, cis-acting element analysis revealed the presence of multiple MeJA-responsive elements in the promoters of *SiSK2*, *SiSK3* and *SiSK8*, corroborating the quantitative expression data. Collectively, these results indicate that *SiSK5*, *SiSK6*, *SiSK7* and *SiSK8* are likely involved in the MeJA response in foxtail millet, whereas the functional relevance of *SiSK1*, *SiSK2*, *SiSK3*, *SiSK4* and *SiSK9* warrants further investigation.

## 4 Discussion

SHAGGY-like kinases are key regulatory factors that are extensively involved in a wide range of physiological and biochemical processes, such as cell growth, tissue development, and stress responses. Despite the extensive research on SHAGGY-like kinases in model plants, their functions in foxtail millet are still poorly understood. This study characterize the *SiSKs* and explore its potential regulatory roles in growth, development, and environmental adaptation. The results demonstrate that although the nine identified SiSKs possess typical conserved protein domains and structural motifs, along with similar secondary and tertiary structures, they exhibit significant evolutionary diversification. Furthermore, *SiSKs*, which possess a rich array of hormone related cis-elements in their promoter region, are highly responsive to multiple abiotic stresses. Transcriptome analysis revealed that *SiSKs* are expressed across various tissues and developmental stages in foxtail millet, corroborating their pivotal function throughout the plant’s growth and development.

The findings regarding abiotic stress responses in foxtail millet align with the established functions of SHAGGY-like kinases in model species. In rice, *OsSK41* has been shown to contribute to salt tolerance (Thitisaksakul et al., 2017a; b), while in maize, *ZmSK3* participates in the regulation of salt tolerance (Li et al., 2023). In this study, phylogenetic analysis revealed that *SiSK8* shares close homology with *OsSK41* and *ZmSK3*, prompting speculation that *SiSK8* may possess similar functions. This hypothesis was preliminarily corroborated by quantitative analysis, which demonstrated that *SiSK8* expression underwent significant alterations within 24 hours of salt treatment. Similarly, *OsSK21* and *ZmSK1* are known to regulate salt and drought tolerance in rice and maize, respectively (Koh et al., 2007; Liu et al., 2025; Xiang et al., 2025). Given their close homology to *SiSK6* and the significant changes in *SiSK6* transcript levels observed under drought and salt stress, we posit that *SiSK6* plays a critical role in modulating foxtail millet stress responses. Likewise, *ZmSK4* is functionally analogous to *ZmSK3* in regulating maize salt tolerance (Li et al., 2023); our data indicate that *SiSK1* is phylogenetically proximate to *ZmSK4* and exhibits significantly reduced expression under salt stress, strongly suggesting its involvement in salt tolerance.

In addition to their important regulatory roles in abiotic stress, some studies have found that the SHAGGY-like kinase gene family is also involved in regulating grain size (*OsSK41*), leaf development, floral organ development (*AtSK12*), embryogenesis (*ZmSK2*), and root growth (*AtSK11,AtSK12,OsSK22*) (Sun et al., 2015; Hu et al., 2018; Chen et al., 2020; Dong et al., 2020; Lyu et al., 2020; Hou et al., 2022; Wang et al., 2022; Zhou et al., 2025), demonstrating the functional diversity of the SHAGGY-like kinase gene family during plant growth and development. Phylogenetic analysis revealed high homology between *OsSK41* and *SiSK8*, as well as between *ZmSK2* and *SiSK4*. Furthermore, expression profile analysis indicated that *SiSK4* and *SiSK8* are highly expressed during inflorescence and seed development. Consequently, we speculate that *SiSK4* and *SiSK8* likely play regulatory roles in the reproductive development of foxtail millet. Investigations into the role of SHAGGY-like kinases in plant growth and development also provide novel insights for exploring the functional characteristics of *SiSKs*.

Crucially, in these studies on the regulation of plant growth and development, members of the SHAGGY-like kinase gene family exert their regulatory effects on development primarily through the brassinosteroid (BR) signaling pathway. In *Arabidopsis thaliana*, the AtSK member *BIN2/SK21* serves as a central negative regulator within the BR signaling pathway and represents the most extensively studied GSK3 homolog in plants (Li and Nam, 2002). Furthermore, several OsSKs have been demonstrated to function as negative regulators of BR-dependent gene expression by phosphorylating and modulating the activities of multiple transcription factors involved in this pathway (Tong et al., 2012; Gao et al., 2019). Then, do the *SiSKs* regulate the growth and development of foxtail millet through the BR pathway? This requires more indepth follow-up research.

## 5 Conclusions

In conclusion, this study establishes a foundational genomic and expression-based framework for the SiSKs, highlighting its structural diversification and pivotal role in abiotic stress responses. To further elucidate the biological functions of the SiSKs, we will conduct comprehensive functional investigations in future work. Firstly, the preliminary functional insights obtained from RT-qPCR validation under five stress treatments should be expanded through stable genetic transformation. This involves isolating the core coding sequences of representative SiSK members and performing heterologous expression in model plants such as *Arabidopsis thaliana* and *Oryza sativa* to definitively elucidate their biological functions. Furthermore, we can generate overexpression and CRISPR/Cas9-mediated knockout transgenic foxtail millet lines to further characterize the functions of the SHAGGY-like kinase genes and dissect their underlying molecular mechanisms. In conclusion, the systematic identification and expression profiling of the SiSKs conducted in this study provides a critical foundation for deciphering the complex regulatory networks governing growth and stress resilience in C4 model crops.

## Data Availability

The original contributions presented in the study are included in the article/**Supplementary Material**. Further inquiries can be directed to the corresponding author.
