# Supplementary material for: Genome-wide identification and expression analysis of the SHAGGY-like kinase gene family in foxtail millet (Setaria italica L.)
Source: Front Plant Sci. 2026 Jun 1;17:1852233. doi: 10.3389/fpls.2026.1852233 (PMC13265537; doi:10.3389/fpls.2026.1852233)
Supplement: Supplementary file 1 [file DataSheet1.docx]

Supplementary Material

# Supplementary Data

Table. S1 Primers used for RT-qPCR analysis in this study

| **Gene** | **Specific Primer** |
| --- | --- |
| *SiSK1* | F:5’- AGATTTTCCACAAGCGGAT-3’ |
|  | R:5’- CTTCAAGAGCAGTGCATCGT-3’ |
| *SiSK2* | F:5’- CCTTAATTTGGTGCTTGAGT-3’ |
|  | R:5’- GAATGTATGCCAAAGCTCT-3’ |
| *SiSK3* | F:5’- CCTCCTCACGGTCACGCTA-3’ |
|  | R:5’- AGGTCCACTCCCAAATAAAGAAT-3’ |
| *SiSK4* | F:5’- TTACCTCAATTTGGTGCTT-3’ |
|  | R:5’- TGACATATATCAACGGCAT-3’ |
| *SiSK5* | F:5’- TGCCACAGAGATATCAAGC-3’ |
|  | R:5’- GACCAGAACTTTTGCACT-3’ |
| *SiSK6* | F:5’- GCACTACAGCAATGCGAACCAG-3’ |
|  | R:5’- TTTACGTCCCTGTGGCAGACTCC-3’ |
| *SiSK7* | F:5’- GACATTAAGCCGCAAA-3’ |
|  | R:5’- TTTTACCAGAACTTTCGC-3’ |
| *SiSK8* | F:5’- TCCCGCAAATTAAAGCTC-3’ |
|  | R:5’- CCTGCTAACAAGATCCAC-3’ |
| *SiSK9* | F:5’- TGCCAAATACTACAATCGG-3’ |
|  | R:5’- TGAGGCTTAATATCACGATG-3’ |
| *SiActin* | F:5’- CGCATATGTGGCTCTTGACT-3’ |
|  | R:5’- GGGCACCTAAATCTCTCTGC-3’ |

**All primers were designed using Oligo7 software.**

**Table. S2 Information on GSK3 Gene Family Members in the Four Investigated Species**

| **Latin name of specie** | **Gene name** | **Gene ID** |
| --- | --- | --- |
| *Arabidopsis thaliana* | AtSK11 | AT5G26751.1 |
|  | AtSK12  AtSK13  AtSK21  AtSK22  AtSK23  AtSK31  AtSK32  AtSK41  AtSK42 | AT3G05840.1  AT5G14640.1  AT4g18710.1  AT1g06390.1  AT2g30980.1  AT3g61160.1  AT4g00720.1  AT1g09840.1  AT1g57870.1 |
| *Oryza sativa* | OsSK11 | LOC_ Os01g14860.1 |
|  | OsSK12  OsSK13  OsSK21  OsSK22  OsSK23  OsSK24  OsSK31  OsSK41 | LOC_ Os01g19150.1  LOC_ Os05g04340.1  LOC_Os01g10840.1  LOC_ Os05g11730.1  LOC_ Os02g14130.1  LOC_ Os06g35530.1  LOC_ Os10g37740.1  LOC_ Os03g62500.1 |
| *Zea mays* | ZmGSK1 | Zm00001d008893 |
|  | ZmGSK2  ZmGSK3  ZmGSK4  ZmGSK5  ZmGSK6  ZmGSK7  ZmGSK8  ZmGSK9  ZmGSK10  ZmGSK11 | Zm00001d009055  Zm00001d012869  Zm00001d016188  Zm00001d024729  Zm00001d029664  Zm00001d037010  Zm00001d039407  Zm00001d040263  Zm00001d048564  Zm00001d053548 |
| *Triticum aestivum* | TaSK11-3A  TaSK11-3B  TaSK11-3D  TaSK12-4A | TraesCS3A02G164200.1  TraesCS3B02G201800.2  TraesCS3D02G177600.2  TraesCS4A02G361100.1 |
|  | TaSK12-5B  TaSK12-5D  TaSK13-1A  TaSK13-1B  TaSK13-1D  TaSK21-3A  TaSK21-3B  TaSK21-3D  TaSK22-1A  TaSK22-1B  TaSK22-1D  TaSK31-1A  TaSK31-1B  TaSK31-1D  TaSK41-1A  TaSK41-4A  TaSK41-5B  TaSK41-5D | TraesCS5B02G511800.3  TraesCS5D02G512300.1  TraesCS1A02G077200.2  TraesCS1B02G095200.2  TraesCS1D02G079300.1  TraesCS3A02G136500.1  TraesCS3B02G208800.1  TraesCS3D02G137200.1  TraesCS1A02G125000.1  TraesCS1B02G142300.1  TraesCS1D02G129000.2  TraesCS1A02G189000.1  TraesCS1B02G196700.1  TraesCS1D02G187400.1  TraesCS1A02G407700.4  TraesCS4A02G345500.2  TraesCS5B02G529300.1  TraesCS5D02G528400.1 |
